# Aerosol Thermodynamics: Nitrate Loss from Regulatory PM_2.5_ Filters in California

**DOI:** 10.1021/acsestair.3c00013

**Published:** 2023-11-29

**Authors:** Yin Ting T. Chiu, Annmarie G. Carlton

**Affiliations:** Department of Chemistry, University of California, Irvine, Irvine, California 92697, United States

**Keywords:** air quality, surface monitors, PM_2.5_, nitrate, NAAQS attainment

## Abstract

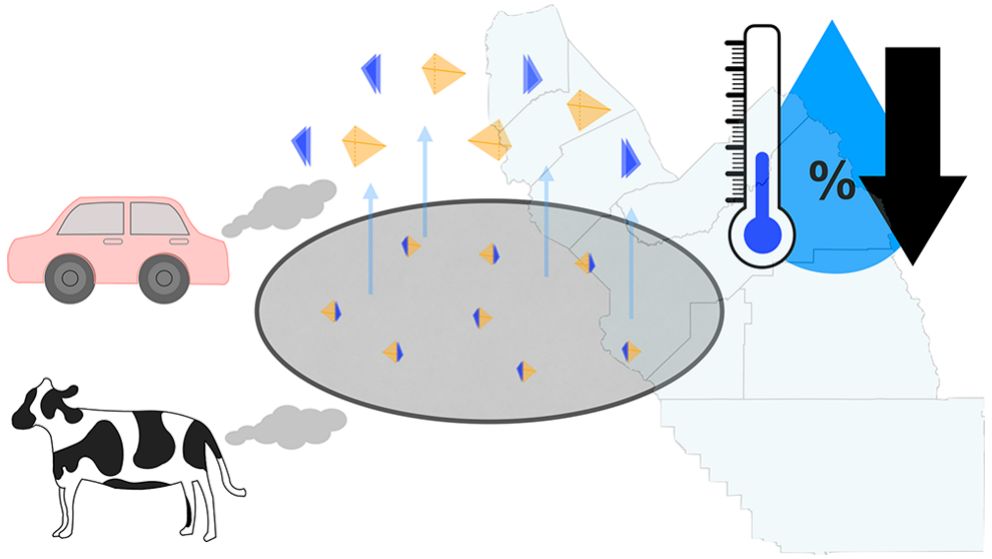

Fine particulate matter (PM_2.5_) mass concentrations reported by regulatory networks are declining across the United States. It is well established that ammonium nitrate contributes substantially to the PM_2.5_ mass in the western United States, and that Teflon filters commonly used by regulatory monitors are subject to negative mass artifacts due to ammonium nitrate volatilization. This study focuses on the San Joaquin Valley (SJV), an environmental justice (EJ) and agricultural region with persistently poor air quality. The SJV is a serious nonattainment area of PM_2.5_ National Ambient Air Quality Standards (NAAQS) with substantial nitrate mass concentrations. We explicitly model the chemical thermodynamic equilibrium of the ammonium nitrate–nitric acid systems and quantify volatilization across California as a function of the deliquescence point relative humidity (%DRH). Nitrate loss is estimated at all federal reference method (FRM) and federal equivalent method (FEM) monitors from 2001 to 2021. Nearly 20% of PM_2.5_ mass is lost from filters in the SJV area, especially during winter and fall when particulate nitrate mass is most abundant. All decadal PM_2.5_ trends calculated from reported measurements in Kern, Tulare, and Fresno counties in the SJV show greater decline in PM_2.5_ mass when nitrate loss is accounted for, up to a factor of 20 in Kern county. This suggests PM_2.5_ mass concentrations reported in regulatory networks are biased low relative to the actual atmospheric burden, notably in an EJ area that lags behind most of the country’s air quality improvements.

## Abstract 2

This work estimates loss of the PM_2.5_ chemical constituent nitrate from surface air quality monitors used in attainment assessments with a focus on California.

## Introduction

The United States Environmental Protection Agency (USEPA) regulates fine particulate matter (PM_2.5_) with a National Ambient Air Quality Standard (NAAQS).^1^ PM_2.5_ exposure is associated with cardiovascular disease, respiratory illness, and mortality, and is a global health burden.^2−6^ State and local entities measure surface mass concentrations of PM_2.5_ as part of an EPA network with federal reference and federal equivalent methods (FRM and FEM). USEPA’s chemical speciation network (CSN), implemented in 2001, chemically characterizes PM_2.5_ and is a helpful tool to quantitatively understand sources and associations among ambient PM_2.5_ chemical composition, health end points, and visibility degradation in urban areas.^7^ Since the introduction of the CSN in 2001 through 2021, PM_2.5_ mass concentrations have declined by 37% across the United States.^8^ This trend is largely a co-benefit of EPA rules to reduce acidic deposition and surface ozone mixing ratios through emission reductions of SO_2_ and NOx (NO + NO_2_) respectively.^9−11^ These pollutants are also precursors of ammonium sulfate and ammonium nitrate and are substantial contributors to ambient PM_2.5_.^12^ In contrast, ammonia, also a PM_2.5_ precursor, is not similarly regulated, and mixing ratios are rising, predominantly in areas with large animal husbandry enterprises such as the San Joaquin Valley (SJV) and the Midwest.^13−15^ The 37% decrease in PM_2.5_ is a national average, driven in part by sulfate and nitrate, and not all communities experience improvement.^16−18^

PM_2.5_ chemical constituents have different physicochemical properties. Both ammonium sulfate and ammonium nitrate are hygroscopic and promote water uptake. Sulfate is not volatile,^19−21^ while it is well established that ammonium nitrate is volatile and evaporates during filter-based PM_2.5_ measurement.^22−26^ This is primarily due to changes in the ambient temperature and the pressure drop across the filter during sampling. In particular, PM_2.5_ FRM and FEM techniques employed in EPA regulatory networks and used for attainment decisions predominantly measure PM_2.5_ mass with polytetrafluoroethylene (PTFE) Teflon and glass fiber filters that generally exhibit negative artifacts for nitrate mass (Table 1).^7,27−32^ Nitrate loss is documented in the literature for PTFE Teflon filters,^7,23−29,33−36^ glass fiber filter tape,^22,37,38^ and Teflon-coated borosilicate glass fiber.^39,40^ Glass fiber filters used in the Met One BAM-1020 FEM are estimated to have greater ammonium nitrate volatilization loss than from Teflon filters.^22,37^ Studies demonstrate that this FEM monitor exhibits both positive and negative artifacts for PM_2.5_ mass due to temperature, liquid water, and acidic gas absorption.^41^ The FEM tapered element oscillating microbalance with a filter dynamic measurement system (TEOM-FDMS) employs a Teflon-coated glass fiber filter and accelerates nitrate loss due to the heating of filters during sampling.^39,40^

**Table 1:** Filter Types of Designated Reference and Equivalent Methods Used in This Study

filter type	FRM/FEM samplers that use this filter
PTFE Teflon	Graseby Andersen Model RAAS2.5-300 Sequential
	Met One Instruments, Inc. E-SEQ-FRM
	Rupprecht & Patashnick Partisol-FRM Model 2000
	R&P Partisol-Plus Model 2025 Sequential
Nafion membrane (PTFE backbone)	Grimm Model EDM 180 PM_2.5_ Monitor
	Thermo Scientific FH62C14-DHS Continuous
Pallflex TX40 (Teflon-coated borosilicate glass fiber)	Thermo Scientific TEOM 1400a with Series 8500C FDMS
	Thermo Scientific TEOM 1405-F with FDMS
glass fiber filter tape	Met One BAM-1020 Beta Attenuation
	Met One BAM-1022 Real Time Beta Attenuation
	Teledyne Model 602 BetaPLUS
	Teledyne Model T640 (with 640X option)
	Thermo Scientific Model 5014i

All measurements are operationally defined, and EPA defines PM_2.5_ measurements in Title 40 of the Code of Federal Regulations (CFR 40) Part 50 Appendix L that specifies that the FRM employs polytetrafluoroethylene (PTFE) Teflon filters (46.2 mm diameter ± 0.25 mm, 2 μm pore size, 30–50 μm filter thickness). Ionic PM_2.5_ constituents, such as sulfates and nitrates, are separately measured as part of the CSN with nylon filters and nitric acid denuders (Table S1).^27,29,42,43^ Teflon filters are known to be susceptible to negative sampling artifacts for nitrate.^23,29,33,35^ Ammonium nitrate vapor pressure increases with temperature and drives evaporation, relative to the partial pressure in the atmosphere. Volatilization from Teflon filters can be quantified from the ambient temperature, amount of particulate nitrate, PM_2.5_ mass, and understanding of relative humidity (RH). In comparison, nylon filters are less prone to ammonium nitrate volatilization and measure nitrate concentrations that more accurately describe ambient concentrations as nylon filters retain nitrate in the form of nitric acid well.^27,28,34^ Other studies show loss of ammonium nitrate due to additional confounding factors such as adsorption of nitric acid by aluminum surfaces in monitors, and sample matrix effects, including particle phase reactions on filters with strong acids such as hydrochloric acid (HCl) and sulfuric acid (H_2_SO_4_).^26,44−46^ State of the art PM_2.5_ measurement techniques such as the Time-of-Flight Aerosol Chemical Speciation Monitor (ToF-ACSM) are in good agreement with FRM/FEM, however, previous literature suggests that particles that undergo drying in inlets of aerosol mass spectrometers (AMS or Q-AMS) are also subject to losses during sampling.^47,48^ Moreover, the presence of organic compounds can interfere with nitrate detection using the Q-AMS and standard ACSM.^49^ High-resolution AMS and ACSM are not subject to these interferences.^50,51^

In this work, we focus on the fundamental equilibrium chemistry that drives the evaporative losses of nitrate in PM_2.5_ from filter-based methods. For condensed phase ammonium nitrate in equilibrium with gaseous nitric acid and ammonia, the ratio between the gaseous and condensed phase compounds is defined by the dissociation constant, *K*_amb,*i*_. The dissociation constant is dependent on the deliquescence relative humidity (%DRH), which is a function of ambient temperature. %DRH for ammonium nitrate is 62% at 25°C.^45,52,53^ Previous research in arid locations to estimate nitrate loss from Teflon filters used in regulatory networks employs a constant factor and does not account for the change in *K*_amb,*i*_ above the %DRH.^27,29^ Constant factors obscure accurate understanding of nitrate loss and the true ambient PM_2.5_ burden, and subsequently, the effectiveness of control strategies in different communities and associations with health end points. Previous research demonstrates that PM_2.5_ is a function of liquid water and that loss from filters has a RH dependence; therefore, we consider the aerosol phase state to account for different dissociation constant values at ambient temperature during each hour when above and below %DRH conditions.^35,36,38,45^

The top three most polluted cities in the U.S. for year-round particle pollution in 2022 are Bakersfield, Fresno, and Visalia, located in Kern, Fresno, and Tulare counties, respectively.^54^ All three counties are a part of the SJV in central California, where nitrate is a dominant constituent of PM_2.5_.^47,55^ The SJV is a critically important and highly productive agricultural region in the United States, especially for the dairy and cattle industries^56^ that are major sources of the area’s particulate nitrate. California defines most of the SJV as disadvantaged communities (SB 535).^57^ The annual average PM_2.5_ concentrations for Kern, Fresno and Tulare counties between 2018–2020 are 17, 15, and 16 μg m^–3^ respectively, and all exceed the annual PM_2.5_ NAAQS that was 15 μg m^–3^ from 1997–2012 and 12 μg m^–3^ since 2012.^1,58^ PM_2.5_ concentrations in counties of the SJV area consistently exceed NAAQS standards and filter measurements are prone to ammonium nitrate losses during measurement in this dry and hot climate.^59^ There is no clear strategy to improve air quality in this region. A recent EPA proposed rule plans to reject the California State Implementation Plan (SIP) for the SJV to attain the PM_2.5_ NAAQS, in part due to uncertainty related to agricultural emissions that form particulate nitrate.^60^ There is currently no approved plan to bring SJV into attainment. Previous studies demonstrate that across the U.S., PM_2.5_ ammonium nitrate volatilization is the greatest in California.^28^ Therefore, we investigate how reported PM_2.5_ mass concentration may be biased low in the San Joaquin Valley due to substantial losses of a dominant chemical constituent. Gaps among regulatory requirements and scientific understanding may contribute to the region’s persistently poor air quality.

## Methods

### Data Collection and Calculations

PM_2.5_, sulfate, nitrate mass concentrations, and meteorology data between 2001 and 2021 are obtained from the EPA Air Quality System (AQS), which includes data from the FRM/FEM and CSN.^61^ PM_2.5_ is obtained from the FRM/FEM, while sulfate and nitrate mass concentrations are obtained from the CSN. We analyze annual average and annual seasonal average trends, where winter consists of January, February, and December months of the same year, spring consists of March, April, and May months, summer consists of June, July, and August months, and fall consists of September, October, and November months. Table 1 shows the types of filters used in FRM/FEM samplers and included in this analysis where nitrate loss is documented in the literature.

There is specific focus on data obtained from 3 CSN sites and 7 FRM/FEM sites in Kern County and Fresno County, 3 CSN and 2 FRM/FEM sites in Tulare county, and 3 CSN sites and 14 FRM/FEM sites in Los Angeles County. It should be noted that the CSN provides reconstructed PM_2.5_ mass, which varies in comparison with FRM/FEM-measured PM_2.5_.^62^ Site locations analyzed here are provided in Figure S2. PM_2.5_ and its chemical constituents are typically measured every 1-in-3 days; some sites sample with daily or every 1-in-6 days frequency. All reported negative concentration values are set to zero, and this may indicate concentrations measured near or below the limit of detection. Hourly surface temperature and relative humidity are obtained from USEPA meteorological data from weather stations across the contiguous United States (CONUS) and aggregated, by mean and median respectively, for each site monitor location.

Teflon filter-collected nitrate losses are calculated based on the thermodynamic equilibrium between the vapor pressure product of ammonium nitrate (particulate nitrate, pNO_3_) and the product of gas-phase nitric acid (HNO_3_) and ammonia (NH_3_) partial pressures (eq 1):^27,28,53^
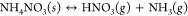
1Hering, Cass, and colleagues applied eq 2 to ambient samples to calculate the daily average nitrate losses (ΔNO_3_) in micrograms per cubic meter (μg m^–3^) from the conversion of thermodynamic equilibrium constants (eq 2). Assumptions include that ammonia and nitric acid are formed in equal parts from the dissociation of ammonium nitrate, and conditions were below the deliquescence point of NH_4_NO_3_.^27,29^ They were able to characterize nitrate losses from Teflon filters observed in real-time samples collected in southern California accurately, under the assumption that
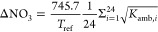
2

The reference temperature, *T*_ref_, is the daily mean temperature calculated from each *i*th hour value. *K*_amb,*i*_ is the dissociation constant calculated at ambient temperature at each hour, *T*_*i*_, from eq 3. It is important to consider the changes in *K*_amb,*i*_ throughout the day because volatilization is a function of temperature and RH, which also varies throughout the day (Figure S3). The partial pressure of gas-phase nitric acid in nanobars (nb) is converted to particle-phase nitrate concentrations (μg m^–3^) with the conversion factor of 745.7 for *T*_ref_.^27,53^ Below %DRH, the thermodynamic equilibrium dissociation constant, *K*_amb,*i*_ (nb^2^) is calculated from a Van’t Hoff equation derived by Mozurkewich (eq 3):^53^
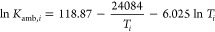
3The dissociation constant derived from eq 3 is applicable to the volatilization of ammonium nitrate below the deliquescence point.

The chemistry that describes the process of ammonium nitrate volatilization depends on whether conditions are above or below the deliquescence point. To determine if conditions are above or below the deliquescence point, we employ eq 4, which is taken from Tang and Munkelwitz:^52^
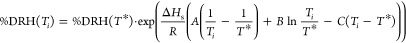
4

The reference temperature (T*) in eq 4 is 298 K, and the thermodynamic and solubility coefficient values for NH_4_NO_3_ are listed in Table 2. Δ*H*_s_ is the integral heat of solution in joules per mole (J mol^–1^), *R* is the gas constant (*R* = 8.3145 J mol^–1^ K^–1^), and *A*, *B*, and *C* are the thermodynamic coefficients that relate solubility to temperature for a single salt solution. Ambient aerosol is a complex mixture, and this simple treatment introduces uncertainty that is difficult to explicitly quantify. When relative humidity is above the deliquescence point of ammonium nitrate, the equilibrium chemistry is more complex^45^ because one must account for acid/base dissociation and the dissociation constant to describe partitioning. *K*_amb,*i*_ in eq 3 is replaced with *K*_amb,i_^*^ to reflect conditions above %DRH:^53^
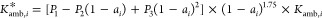
5where *a*_*i*_ is assumed to be the relative humidity as a fraction,^63^ and
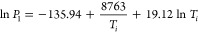
6
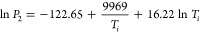
7
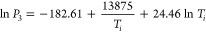
8We evaluate and aggregate hourly ambient temperature (*T*_*i*_) and RH for the nearest FRM/FEM and CSN monitors for the period investigated here to determine if ambient aerosol is above or below the %DRH (eq 4). Individual hours are analyzed for %DRH calculations, and we present the average prevalence for the period of each monitoring location for reference (Figure S4). For the purposes of this study, we assume that (1) particulate nitrate (pNO_3_) measured on nylon filters is the most accurate, as is consistent with previous literature,^34^ and (2) nitrate volatilizes from ammonium nitrate and does not include volatilization from other forms of semivolatile compounds, including organic nitrates. In cases where calculated ΔNO_3_ exceeds the measured pNO_3_, it is assumed that ΔNO_3_ = pNO_3_. This constraint ensures that calculations do not estimate more pNO_3_ on Teflon filters than what would be measured by nylon filters.^29^ We exclude loss of semivolatile organic compounds^64^ in these calculations, and our estimates of ΔNO_3_ are likely a lower bound estimate of the total negative sampling artifacts for PM_2.5_ collected with FRM and FEM monitors. The fraction of PM_2.5_ mass lost by nitrate volatilization (PM_2.5,ΔNO_3__) is calculated with eq 9, where PM_2.5_ mass concentration is obtained from the 24 h FRM:
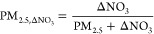
9To test for significance in trends of PM_2.5_, nitrate, and sulfate mass concentrations, we use the Mann Kendall test, a nonparametric statistical test that provides an unbiased Sen slope and is robust against outliers and large data gaps (Table 3).

**Table 2:** Thermodynamic and Solubility Data for NH_4_NO_3_^52^

%DRH at 298 K	Δ*H*_s_ (J mol^–1^)	*A*	*B*	*C*
61.8	16254.84	4.298	–3.623 × 10^–2^	7.853 × 10^–5^

**Table 3:** Mann Kendall Correlation Test in Order of PM_2.5_ Species between 2001 and 2021

county		PM_2.5_	SO_4_^2–^	NO_3_^–^
Los Angeles^a^	Sen slope (μg m^–3^ yr^–1^)	–0.416	–0.176	–0.194
	intercept (μg m^–3^)	18.094	3.375	5.318
	*p*	**<0.05**	**<0.05**	**<0.05**
	trend	decreasing	decreasing	decreasing
Fresno	Sen slope (μg m^–3^ yr^–1^)	–0.293	–0.041	–0.199
	intercept (μg m^–3^)	20.20	1.56	6.28
	*p*	**<0.05**	**<0.05**	**<0.05**
	trend	decreasing	decreasing	decreasing
Tulare^a^	Sen slope (μg m^–3^ yr^–1^)	–0.190	–0.035	–0.182
	intercept (μg m^–3^)	19.765	1.694	6.905
	*p*	0.14	**<0.05**	**<0.05**
	trend	no trend	decreasing	decreasing
Kern^b^	Sen slope (μg m^–3^ yr^–1^)	–0.054	–0.048	–0.216
	intercept (μg m^–3^)	17.565	1.775	6.782
	*p*	0.695	**<0.05**	**<0.05**
	trend	no trend	decreasing	decreasing

a Measurement starts in 2002.

b Missing CSN data from 2014.

## Results and Discussion

**Figure 1:**
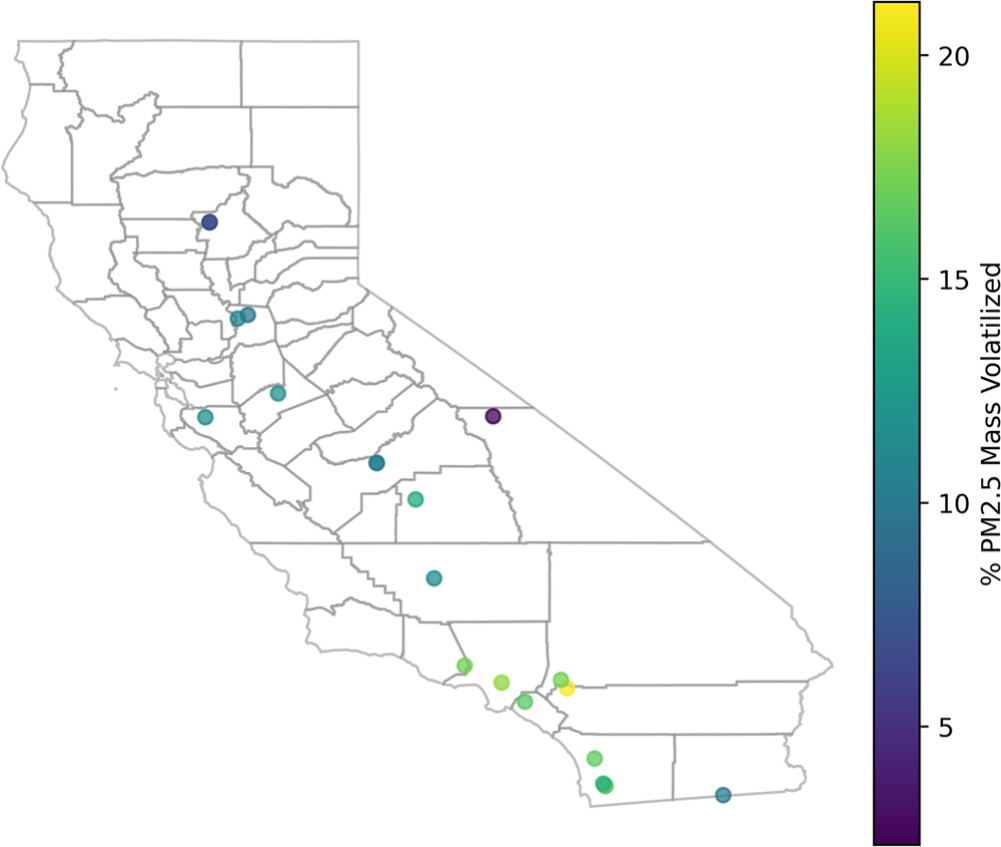
Average percent mass of PM_2.5_ volatilized from Teflon filters used in EPA’s FRM/FEM monitors across California from 2001 to 2021.

PM_2.5_ mass volatilization from surface air quality monitors occurs ubiquitously across California (ΔNO_3_ > 0; Figure 1). The percentage mass of PM_2.5_ volatilized in Teflon filters is greatest in southern California, consistent with previous findings (Figure 1).^28^ In regions with the highest year-round PM_2.5_ mass where nitrate is a dominant chemical constituent (Figure S5) and aerosol is most often under conditions below %DRH (Figure S4), total mass loss is greatest, most notably in winter. We estimate that the monitor in Rubidoux, Riverside County, CA that has an annual average PM_2.5_ concentration of 16.8 μg m^–3^, loses 20.6% of PM_2.5_ mass from Teflon filter measurements used in FRM/FEM monitors, suggesting that the true average ambient burden in Rubidoux is closer to 21.1 μg m^–3^ (Figure 1). We estimate that for other monitor locations in southern California (such as in Los Angeles, Long Beach, and Orange), that measure between 8 and 15 μg m^–3^ annual PM_2.5_ mass, losses are over 15%. The San Joaquin Valley (SJV) averages between 15 and 18 μg m^–3^ of PM_2.5_ and loses between 10 and 15% of PM_2.5_ mass in Teflon filter measurements annually. These findings suggest that the PM_2.5_ mass is likely biased low relative to the actual ambient burden. This is a concern in central and southern California where reported PM_2.5_ mass concentrations are already above the health-based PM_2.5_ NAAQS.

**Figure 2:**
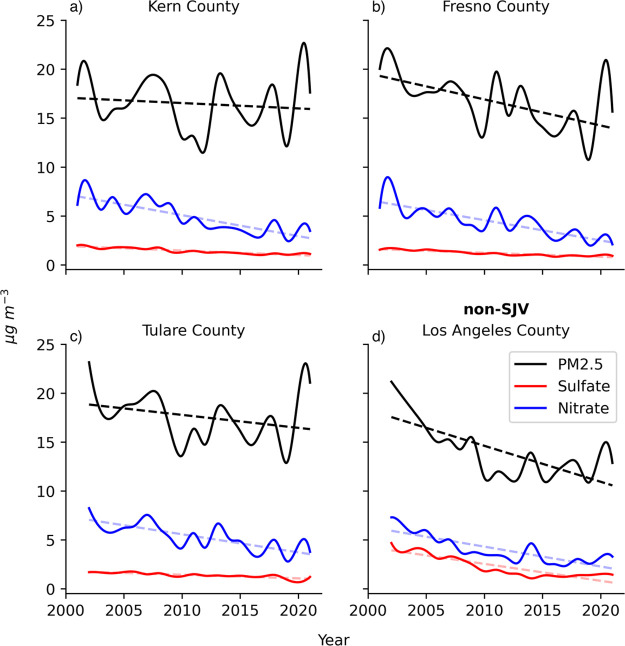
Multidecade trend (μg m^–3^ yr^–1^) in reported annual PM_2.5_ (black), sulfate (red), and nitrate (blue) mass concentrations between 2001 and 2021, for four counties located in the western United States: (a) Kern County (missing CSN data from 2014), (b) Fresno County, (c) Tulare County (measurement starts in 2002), and (d) Los Angeles County (measurement starts in 2002).

PM_2.5_ mass concentrations in 2001 for Kern, Tulare, Fresno, and Los Angeles counties exceeded 15 μg m^–3^ (Figure 2) and were classified as nonattainment in 2005 using the three year rolling averages for PM_2.5_ defined by the NAAQS. Decreasing mass concentrations for PM_2.5_ are significant (p<0.05) for Fresno and Los Angeles counties. Tulare and Kern counties demonstrate no statistically discernible trend for PM_2.5_ mass (Table 3), in sharp contrast to the national average. Air quality in the eastern U.S. is sufficiently improved to now attain PM_2.5_ NAAQS, with one exception in Allegheny county, PA.^65^ Decreasing PM_2.5_ mass trends for the eastern U.S. are driven more heavily by sulfate mass compared to nitrate mass.^66^ Decline in annual average sulfate mass is noted for Los Angeles but not the other California locations analyzed here. The Spearman rank coefficients for PM_2.5_ and nitrate mass are positive and highly correlated at all of the California monitoring sites (Figure S6), consistent with improving air quality due to PM_2.5_ trends in the western U.S. driven by nitrate mass. The SJV and Los Angeles-South Coast Air Basin remain in nonattainment.^65^ Particulate nitrate is a major PM_2.5_ chemical constituent in these locations. In the serious PM_2.5_ nonattainment area of the agricultural SJV, in particular, Tulare and Kern counties, farm workers predominantly spend time in outdoor, ambient environments where the regulatory reported PM_2.5_ mass concentrations are among the least reliable indicators of the true ambient burden. While nationally averaged air quality improved over the last 20 years, these locations lag behind the national average, including other environmental justice areas.

**Figure 3:**
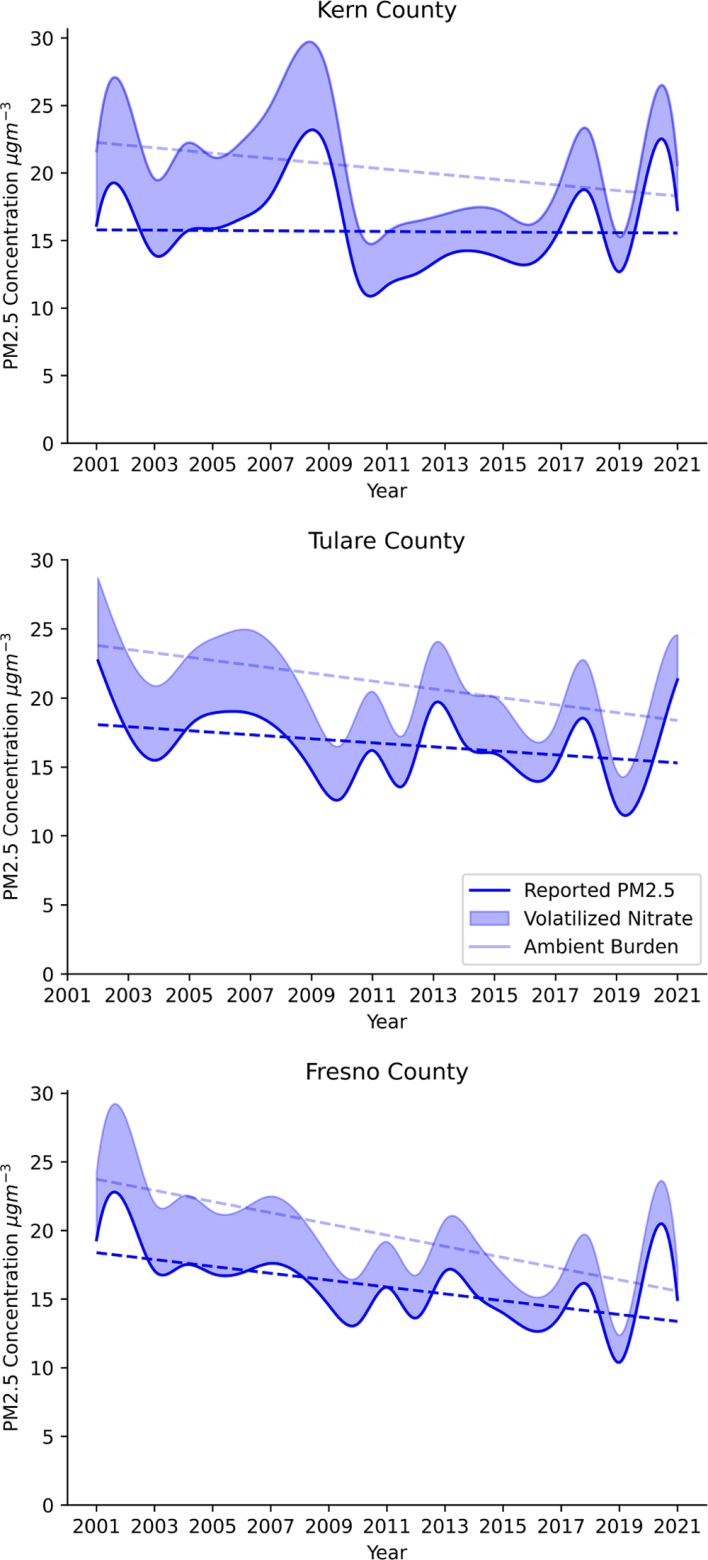
PM_2.5_ reported by the USEPA (solid line) and estimated ammonium nitrate volatilization (shaded region) annually for Kern, Tulare, and Fresno counties.

We explore Kern, Tulare, and Fresno counties in detail because they are environmental justice areas in the SJV where pollution concentrations are regularly monitored and where policy is failing to attain air quality standards that safeguard human health. Reported annual FRM/FEM PM_2.5_ trends show a decline between 2001 and 2021 in Kern, Tulare, and Fresno, with slopes of −0.012, −0.146, and −0.250 μg m^–3^ yr^–1^, respectively (Figure 3). Accounting for nitrate losses, the trends of the ambient burden for Kern, Tulare, and Fresno counties show greater PM_2.5_ decline than reported PM_2.5_, with slopes of −0.199, −0.286, and −0.408 μg m^–3^ yr^–1^, respectively. Annual PM_2.5_ trends in all counties studied are driven by high fall and wintertime particulate nitrate (Figure S7). Volatilized PM_2.5_ from Teflon filters in Kern, Fresno, and Tulare counties are highest in the winter and fall, when concentrations are greatest. Declining coal use and implementation of low sulfur diesel in these regions contribute to decreasing PM_2.5_ mass concentrations.^66^ In the eastern U.S., air quality policy is largely successful in attaining PM_2.5_ NAAQS, where the dominant contributing species is well characterized by regulatory PM_2.5_ monitoring.

Nitrate loss from regulatory monitors confounds interpretation of the true PM_2.5_ burden especially in southern and central California, where frontline communities who typically work in outdoor, ambient environments experience the worst year round PM_2.5_. The discrepancy in reported versus actual annual PM_2.5_ mass for counties located in the western U.S. (Figure S7) is a result of high winter nitrate measurements. Particulate nitrate as a percentage of reported PM_2.5_ mass () is decreasing for the counties studied in California (Figure S8). The sole exception is Tulare county where wintertime  is increasing. Temperatures are highest in the summertime, approaching 30 °C on average, 20 °C in the fall and springtime, and 10 °C in the winter (Figure S9). The SJV has dry summers and more humid winters, with winters becoming drier over the studied period. Hotter and drier conditions promote evaporation in the PM_2.5_ Teflon filters. Summertime PM_2.5_ evaporative losses of nitrate are much lower than wintertime losses; however, this is because higher temperatures result in higher gas phase ammonia and nitric acid that do not condense onto the filter in the first place. It is important to note that ammonium nitrate precursors are abundant at this time, and they are difficult to measure with existing regulatory PM_2.5_ monitors relative to nonvolatile species such as sulfate. As federal reference and equivalent methods of measuring particulate matter remain the standard, there continues to be a loss of PM_2.5_ mass values that are unaccounted for in regulatory networks.

### Conclusion

PTFE filters, as specified by CFR 40 Appendix L, hinder an accurate quantitative understanding of ambient PM_2.5_ mass in some locations. Ammonium nitrate volatilization from PM_2.5_ filters occurs at all EPA FRM/FEM sites across the state of California analyzed here. Reported PM_2.5_ mass in the most serious nonattainment areas is biased low and fails to accurately describe the ambient burden due to volatilization of ammonium nitrate, most notably in southern and central California. While the PM_2.5_ mass is declining on the national level, counties in the SJV do not experience the same level of improvement. Reduction of PM_2.5_ mass is driven by different chemical composition; east coast decadal trends are driven by sulfate concentrations and west coast trends are driven by nitrate concentrations. Reported PM_2.5_ mass concentration in California with high nitrate, low sulfate, and high PM_2.5_ concentrations are most biased due to ammonium nitrate losses in filter measurements. Accounting for nitrate losses, PM_2.5_ mass trends show a greater fractional decline than what is reported by the FRM/FEM, while still remaining in nonattainment of the NAAQS. All studied sites in California continue to exceed PM_2.5_ NAAQS during the time period, indicating that national assessments may continue to neglect some areas, notably the environmental justice communities in the SJV agricultural region. The gap between regulatory definitions and actual burden may contribute to the fact that air quality improvement in the SJV lags behind the national average. In the context of rising ambient temperatures, the ability of FRMs and FEMs to accurately record the pollution burden that adversely impacts human health is an important and outstanding question.

## Supporting Information Available

Additional figures that demonstrate annual, seasonal, and diurnal PM_2.5_, nitrate, and meteorological trends of the areas studied (PDF)
