# Supplementary material for: Aerosol Thermodynamics: Nitrate Loss from Regulatory PM2.5 Filters in California
Source: ACS EST Air. 2023 Nov 29;1(1):25–32. doi: 10.1021/acsestair.3c00013 (PMC10798142; doi:10.1021/acsestair.3c00013)
Supplement: Supplementary file 1 — ea3c00013_si_001.pdf [file ea3c00013_si_001.pdf]

Supporting Information for "Aerosol Thermodynamics: Nitrate Loss from Regulatory $PM_{2.5}$ Filters in California"

Yin Ting T. Chiu and Annmarie G. Carlton*

Department of Chemistry, University of California, Irvine, Irvine, California 92697, United States

E-mail: agcarlto@uci.edu

We provide the code used to calculate nitrate loss values (ΔNO_3) that is publicly available and can be accessed at `https://github.com/yintingchiu/NH4NO3-volatilization/`.

Table S1: Samplers and denuders used to measure CSN nitrate in this study

**Sampler**	**Denuder**
Met One SASS/SuperSASS - Ion Chromatography	Honeycomb (Al/Mg alloy)
Andersen RAAS - Ion Chromatography	Annular (glass)
IMPROVE Module B with Cyclone Inlet	Annular (aluminum)

Table S2: $PM_{2.5}$, SO_4^{2-} and NO_3^- percentage decreases calculated as EPA does in counties between 2001-2021.

County	$PM_{2.5}$ (%)	SO_4^{2-} (%)	NO_3^- (%)
Kern	- 4	- 44	- 43
Tulare†	- 9	- 28	- 54
Fresno	- 22	- 40	- 64
Los Angeles†	- 39	- 69	- 55

†*measurement starts in 2002*

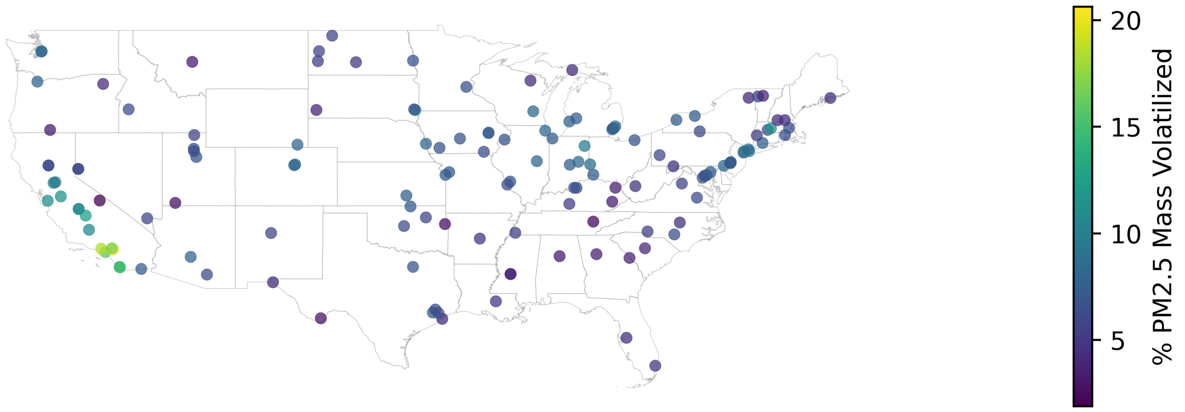

Figure S1: Average percent mass of $PM_{2.5}$ volatilized from Teflon filters used in EPA's FRM/FEM monitors across the CONUS from 2001-2021

Table S3: Average temperature, median RH, changes in temperature and RH between 2001-2021 for four counties

County	Average Temperature (°C)	Median RH	Change in T (°C/year)	Change in RH (%RH/year)
Fresno, CA	17.8	56.6	0.072	-0.187
Tulare, CA	16.1	54.5	0.181	-0.322
Kern, CA	18.3	49.7	-0.013	-0.146
Los Angeles, CA	17.9	65.0	0.041	-0.317

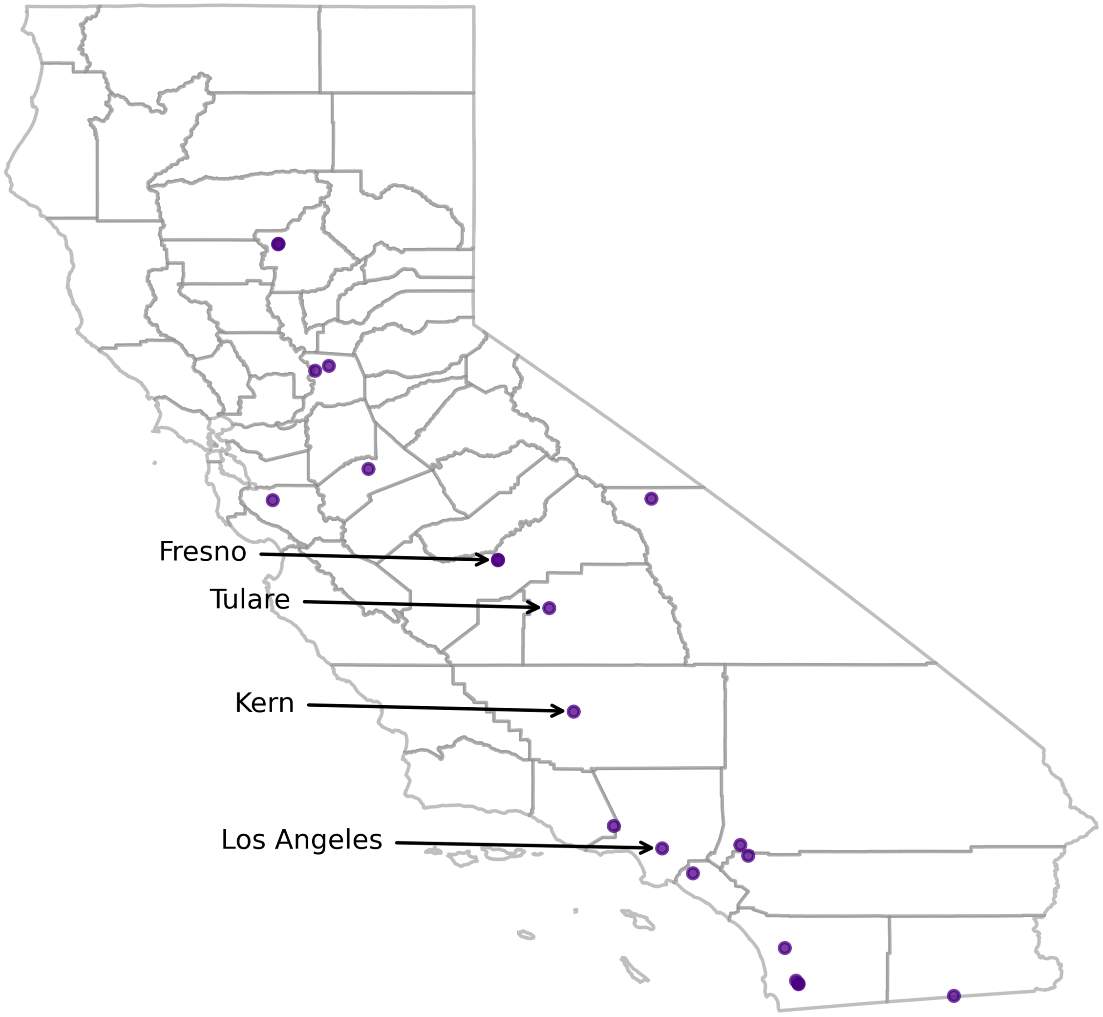

Figure S2: Collocated FRM/FEM and CSN sites, within a minimum range of 4 decimal places in both latitude and longitude that report $PM_{2.5}$ and particulate nitrate, with nearby or collocated weather stations that report hourly relative humidity and temperature across California between 2001-2021

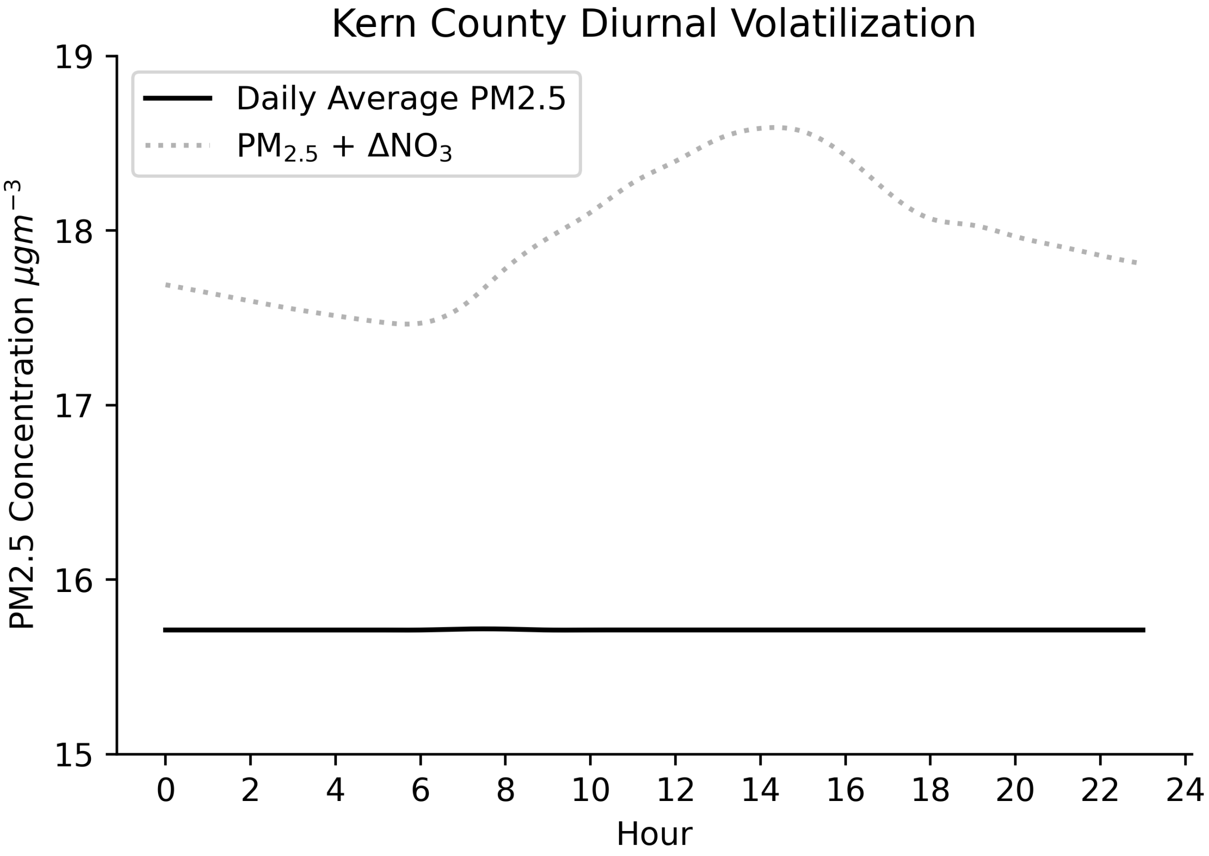

Figure S3: Average diurnal variation of ΔNO_3 in Kern County from 2001-2021 (missing data from 2014), solid line indicates daily average $PM_{2.5}$ as reported by the FRM/FEM

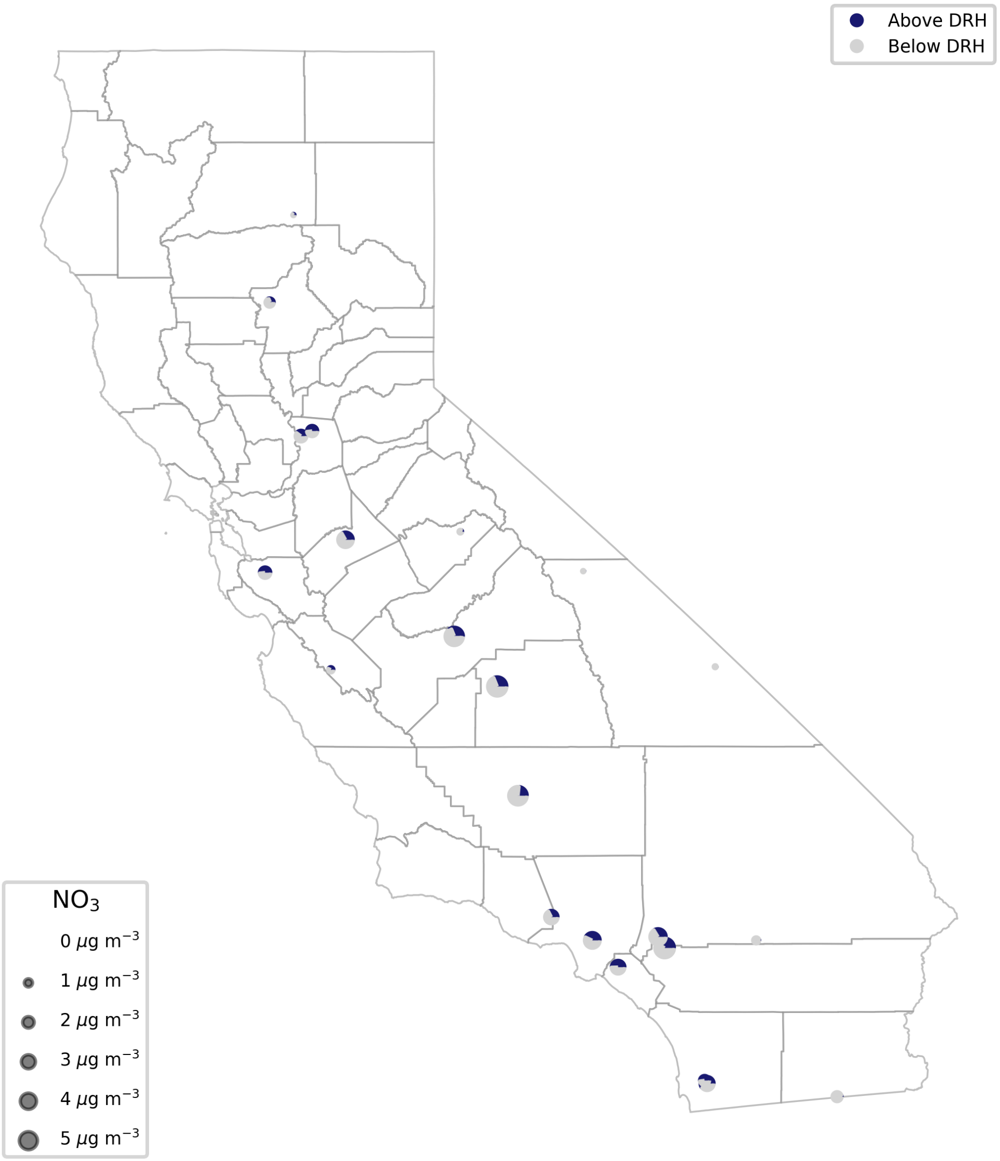

Figure S4: Percentage of datapoints above (blue) and below (grey) %DRH for all CSN sites across California that measured particulate nitrate (size of circle represents average nitrate for the site) between 2001-2021

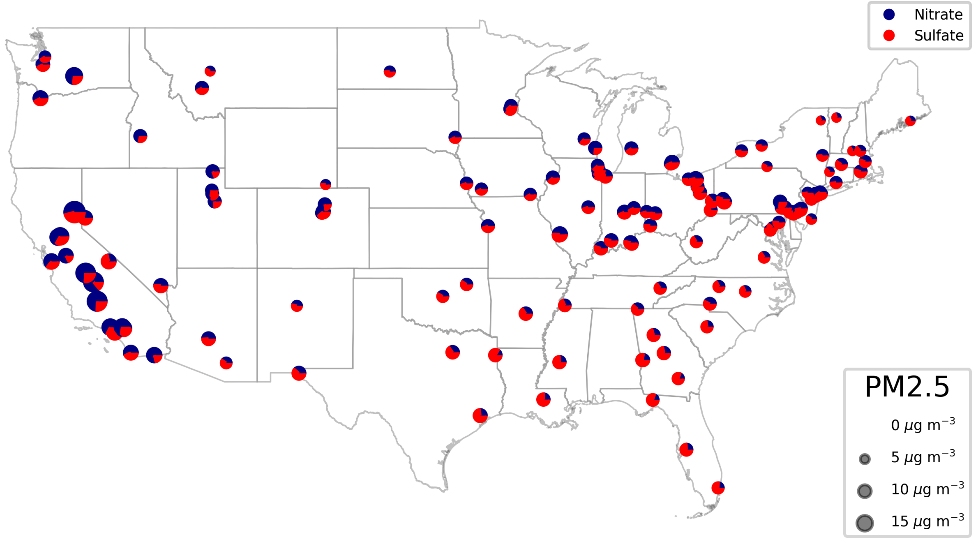

Figure S5: Fractional contribution of nitrate and sulfate anions to their measured sum in $PM_{2.5}$ in 2020

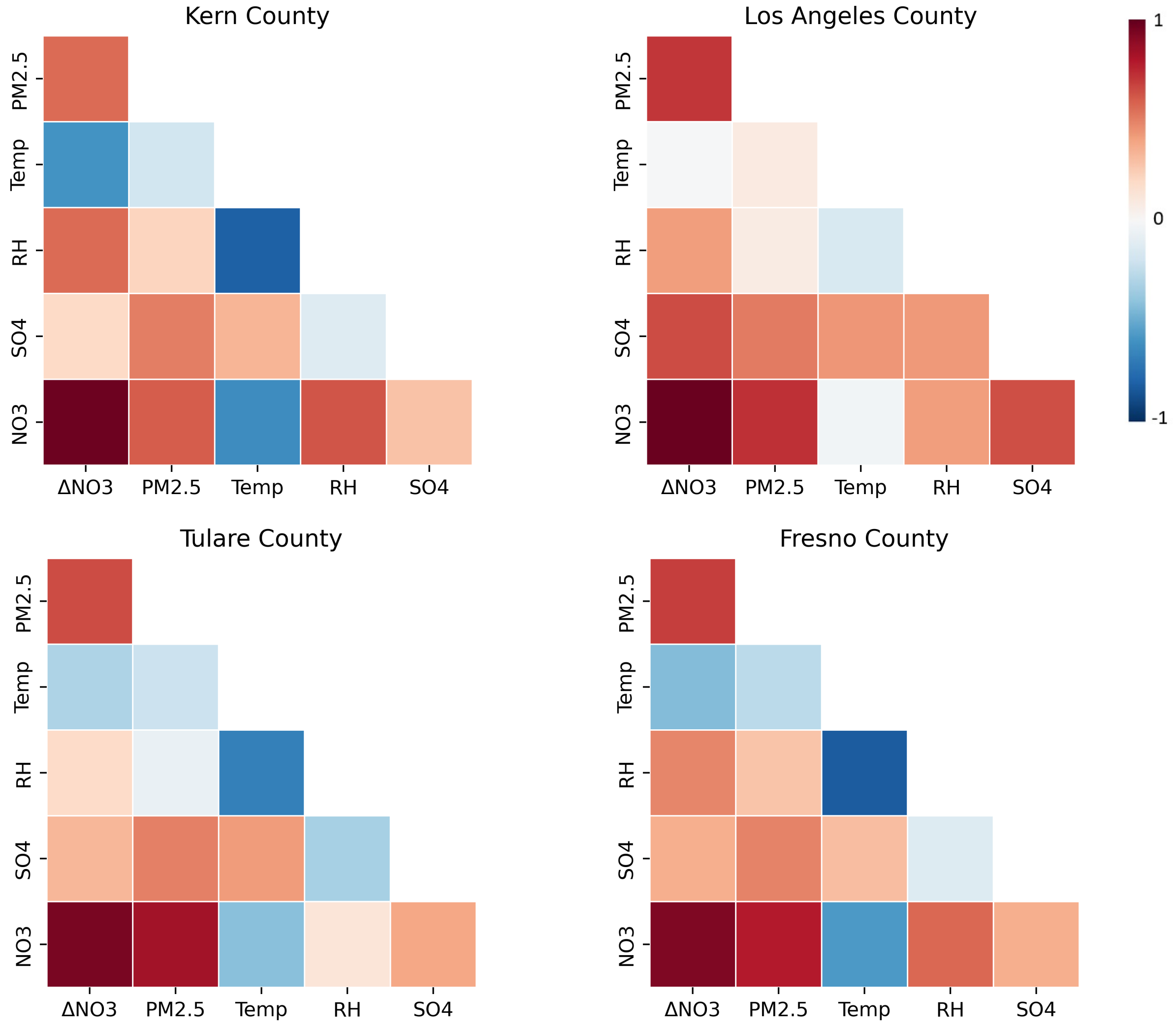

Figure S6: Spearman rank correlation coefficients for ΔNO_3, $PM_{2.5}$, Temperature, Relative Humidity, Nitrate (NO_3) and Sulfate (SO_4) in Kern, Tulare, Fresno, and Los Angeles counties. *Note: ΔNO_3 is calculated and not independent like other variables*

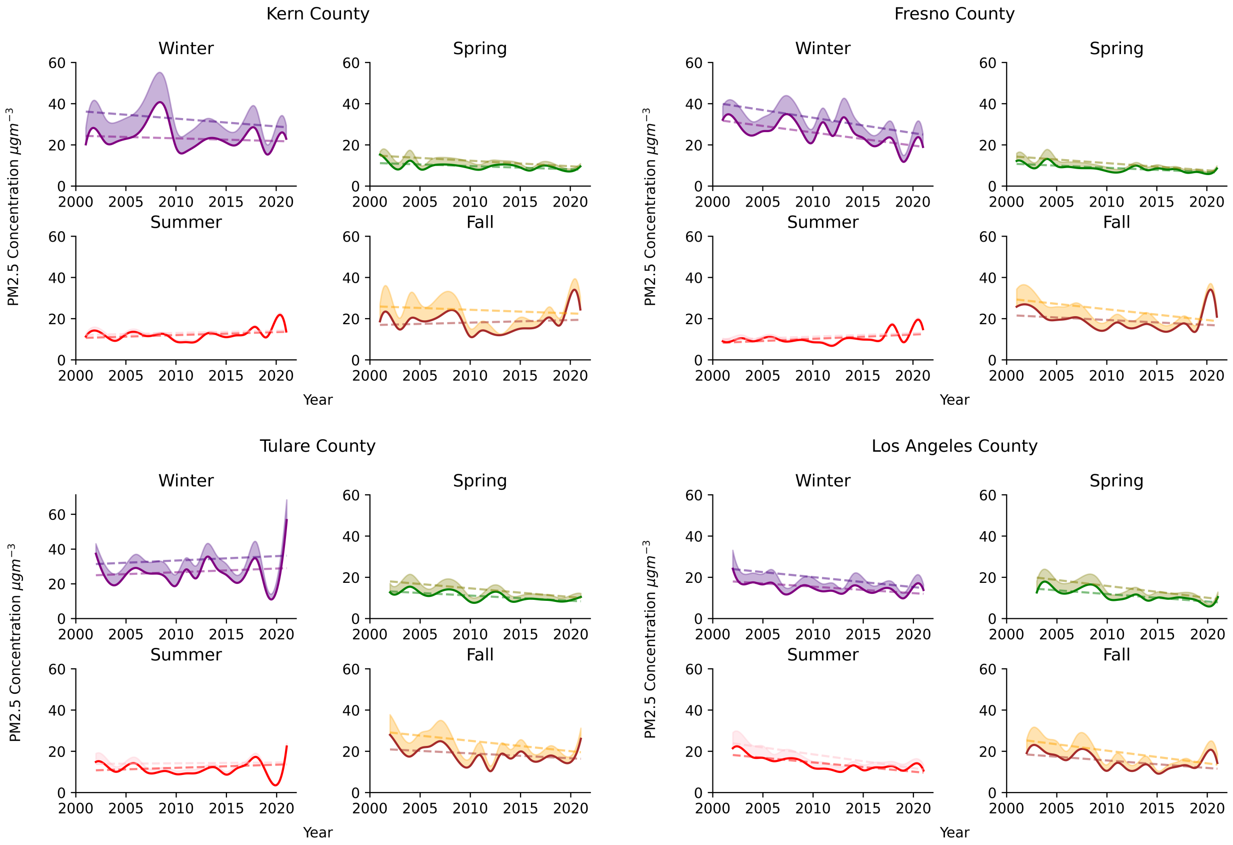

Figure S7: Seasonal $PM_{2.5}$ reported by the USEPA (solid line) and estimated ammonium nitrate volatilization (shaded region) for a) Kern County, b) Fresno County, c) Tulare County, and d) Los Angeles County

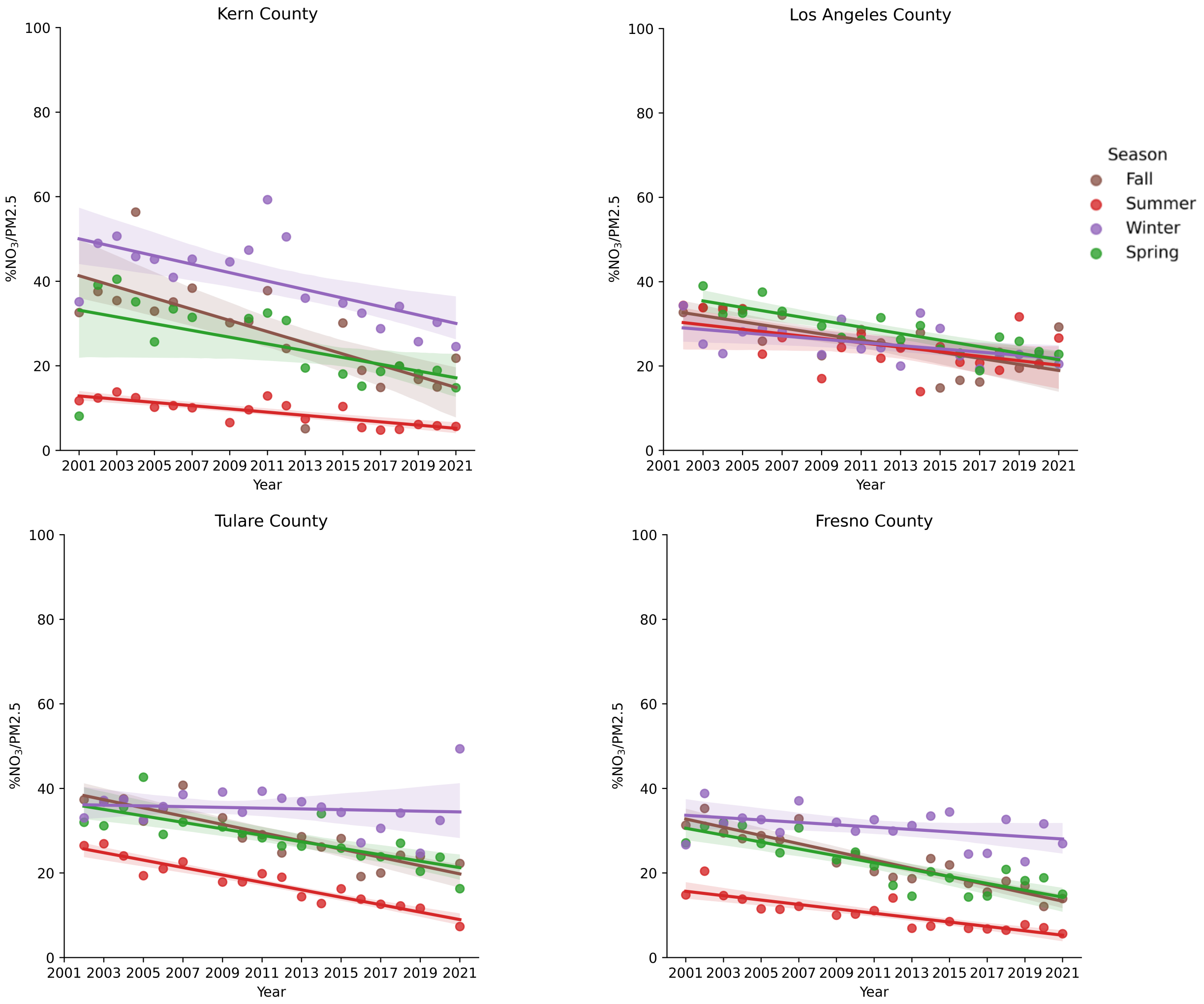

Figure S8: Percentage particulate nitrate in $PM_{2.5}$ mass concentrations as measured by the USEPA CSN for a) Kern County, b) Tulare County, c) Fresno County, and d) Los Angeles County from 2001-2021

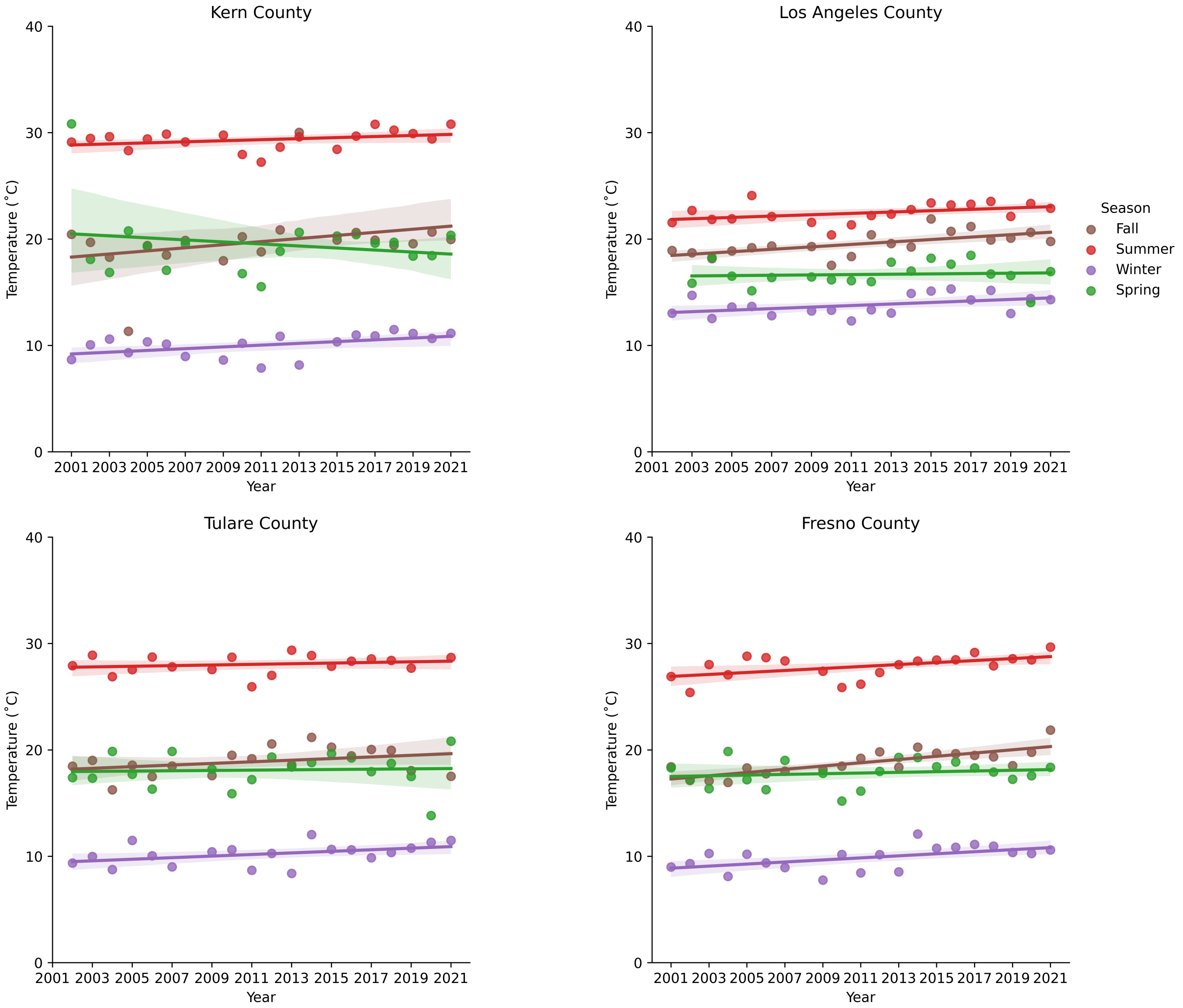

Figure S9: Seasonal temperatures measured by various weather stations for a) Kern County, b) Tulare County, c) Fresno County, and d) Los Angeles County from 2001-2021
